# TMAO enhances TNF-α mediated fibrosis and release of inflammatory mediators from renal fibroblasts

**DOI:** 10.1038/s41598-024-58084-w

**Published:** 2024-04-20

**Authors:** Kapetanaki Stefania, Kumawat Kumar Ashok, Paramel Varghese Geena, Persson Katarina, Demirel Isak

**Affiliations:** 1 School of Medical Sciences, Örebro University, Campus USÖ, 701 82 Örebro, Sweden; 2 Nephrology Department, Karolinska University Hospital, 171 76, Solna, Sweden; 3 Nephrology Department, Karolinska University Hospital, 141 86, Huddinge, Stockholm, Sweden

**Keywords:** TMAO, TNF-α, Renal fibroblasts, Fibrosis, Inflammation, Kidney, Cell biology

## Abstract

Trimethylamine-N-oxide (TMAO) is a gut microbiota-derived metabolite and TNF-α is proinflammatory cytokine, both known to be associated with renal inflammation, fibrosis and chronic kidney disease. However, today there are no data showing the combined effect of TMAO and TNF-α on renal fibrosis-and inflammation. The aim of this study was to investigate whether TMAO can enhance the inflammatory and fibrotic effects of TNF-α on renal fibroblasts. We found that the combination of TNF-α and TMAO synergistically increased fibronectin release and total collagen production from renal fibroblasts. The combination of TMAO and TNF-α also promoted increased cell proliferation. Both renal proliferation and collagen production were mediated through Akt/mTOR/ERK signaling. We also found that TMAO enhanced TNF-α mediated renal inflammation by inducing the release of several cytokines (IL-6, LAP TGF-beta-1), chemokines (CXCL-6, MCP-3), inflammatory-and growth mediators (VEGFA, CD40, HGF) from renal fibroblasts. In conclusion, we showed that TMAO can enhance TNF-α mediated renal fibrosis and release of inflammatory mediators from renal fibroblasts in vitro. Our results can promote further research evaluating the combined effect of TMAO and inflammatory mediators on the development of kidney disease.

## Introduction

Trimethylamine-N-oxide (TMAO) is the product of oxidation of trimethylamine (TMA). This takes place in the liver by the enzyme flavin-containing monooxygenase 3 and by the gut microbiota^1,2^. Organic diet compounds such as choline, betaine, L-carnitine existing in dairy products, eggs, red meat and fish are precursors of TMA and TMAO^1–3^. The kidney is the organ that is primarily responsible for the excretion of TMAO. As a result, kidney function and diet affect the levels of TMAO in blood^1^. TMAO has a physiological role in facilitating the homeostasis of organisms. TMAO acts as a protein stabilizer, a natural osmolyte and an electron acceptor. In the kidneys, TMAO protects urine-concentrating medullary cells from dying due to intracellular accumulation of urea^4,5^.

There is a known association between TMAO and chronic kidney disease (CKD). CKD patients have higher blood levels of TMAO in comparison to non-CKD patients^6^. This is due to decreased glomerular filtration rate (GFR) but also due to increased metabolism of TMA by an altered gut microbiota^7,8^. In CKD patients, gut microbiome alterations occur due to urea accumulation in the gut^9^. Moreover, TMAO is associated with increased cardiovascular risk and all-cause mortality in CKD patients^10–12^.

The renal tubulointerstitium contributes to CKD progression^13^. It consists of tubular cells, fibroblasts, pericytes, immune cells, peritubular endothelium and extracellular matrix^14^. Each of these cell types has a role in initiation and progression of tubulointerstitial inflammation and fibrosis. The process of tubulointerstitial fibrosis includes inflammatory cell infiltration, activation of fibroblasts, production and deposition of extracellular matrix, tubular atrophy and rarefaction of microvasculature^15^. Following kidney injury, inflammatory cells especially lymphocytes, dendritic cells and macrophages, infiltrate the renal interstitium. They become activated and produce reactive oxygen species, fibrogenic growth factors and cytokines^15^. These profibrotic cytokines affect the local microenvironment and induce the phenotypic transition and activation of fibroblasts, tubular cells, endothelial cells and pericytes into myofibroblasts. The majority of the myofibroblasts are derived from interstitial fibroblasts^15^. During the transition of renal fibroblasts to myofibroblasts, a variety of cytokines, chemokines and growth factors are secreted^16^. Tubulointerstitial inflammation and fibrosis lead to oxidative stress, chronic hypoxia, nephron loss, declined renal function and terminal CKD^13,17^.

TNF-α is an inflammatory mediator released from tubular epithelial cells during kidney injury^18^. It is known that TNF-α contributes to renal fibrosis^18,19^. The main signaling pathways activated by TNF-α are those centered around NF-κB and MAPK^18,20^. High renal and serum levels of TNF-α have been found in patients and experimental mouse models with CKD^18^. There is evidence of beneficial effect of anti-TNF-α therapy on renal inflammation and renal function in patients with CKD, especially in those with co-existing rheumatic disease^20^.

There is evidence that TMAO aggravates tubulointerstitial fibrosis and renal inflammation in CKD^6,21–23^. More specifically, TMAO increased tubulointerstitial fibrosis and deposition of collagen in the kidneys of mice^6,21^. Moreover, we have recently shown that TMAO promotes renal fibroblast activation and proliferation *in vitro*^22^.We found that TMAO increases total collagen production and that the effects were mediated by PERK/Akt/mTOR pathway, NLRP3 and caspase-1^22^. Furthermore, TMAO is known to promote renal inflammation and fibrosis in diabetic kidney disease rats^23^. Notably, TMAO contributes to a variety of chronic inflammatory diseases and is associated with vascular inflammation^24,25^. However, to our knowledge, there are currently no studies that have elucidated if TMAO in combination with the fibrotic cytokine TNF-α can enhance renal fibrosis and inflammation. The aim of this study was to investigate whether TMAO can enhance the inflammatory and fibrotic effects of TNF-α on renal fibroblasts.

## Materials and methods

### Culture of human fibroblasts

The human renal medullary fibroblast cell line TK173 was used during all experiments (a kind gift from Professor Anton Jan van Zonneveld, Leiden University, Leiden, The Netherlands)^26^. The renal fibroblasts were cultured in Dulbecco’s modified eagle medium (DMEM) supplemented with 2 mM L-glutamine, 1 mM non-essential amino acids and 10% fetal bovine serum (FBS) (all from Thermo fisher Scientific, Massachusetts, USA) at 37ºC in a 5% CO_2_ atmosphere. The fibroblasts were serum starved overnight in DMEM supplemented with 2 mM L-glutamine and 1 mM non-essential amino acids prior to stimulation. During stimulation, DMEM supplemented with 1% FBS, 2 mM L-glutamine and 1 mM non-essential amino acids was used.

### Stimulation of renal fibroblasts

Renal fibroblasts were stimulated with TMAO (300 µM, Sigma-Aldrich, St. Louis, MO, USA), TNF-α (1, 10 or 50 ng/ml, Sigma-Aldrich) or the combination of both for 24-96 h, depending on the experimental setup, at 37 °C in 5% CO_2_. Renal fibroblasts were pre-stimulated with TMAO for 2 h prior to TNF-α stimulation during the combination treatments. The renal fibroblasts were also pre-incubated with DMSO (vehicle), mTOR inhibitor ridaforolimus (1 µM, Selleckchem, TX, USA), Akt inhibitor MK-2206 (1 µM, Selleckchem), PI3K inhibitor wortmannin (1 µM, Selleckchem) or ERK inhibitor PD98059 (10 µM, Santa Cruz Biotechnology Inc., Heidelberg, Germany) for 1 h prior to TMAO or TNF-α stimulation. Supernatants were collected and stored at -80ºC until further investigation.

### Measurement of fibronectin release and cell viability

Fibronectin release from renal fibroblasts was analyzed using the human fibronectin kit (Duo set, ELISA, R&D Systems, Minneapolis, USA) after 24 h. Cell viability was investigated using the Pierce lactate dehydrogenase (LDH) cytotoxicity assay (Thermo Fisher Scientific) following the manufacturer’s instructions. The Cytation 3 plate reader was used to evaluate the optic density of all assays.

### Proliferation assay

Renal fibroblasts were stimulated with TMAO (300 µM), TNF-α (1, 10 or 50 ng/ml) or the combination of both for 48 h and incubated at 37ºC with 5% CO_2_. Renal fibroblasts were pre-stimulated with TMAO for 2 h prior to TNF-α stimulation during the combination treatments. After stimulation, the renal fibroblasts were washed once with PBS. 0.1% Crystal violet (diluted in 20% methanol, Sigma-Aldrich) was then added to the fibroblasts for 10 min at room temperature and the cells were then washed twice with tap water. The renal fibroblasts were then destained with 1% sodium dodecyl sulfate on a shaker at 500 rpm for 5 min and evaluated at 570 nm using the Cytation 3 plate reader.

### Total collagen production

Renal fibroblasts were stimulated with TMAO (300 µM), TNF-α (1, 10 or 50 ng/ml) or the combination of both in the presence of 50 µg/ml sodium ascorbic acid (Thermo fisher Scientific) for 96 h incubated at 37ºC with 5% CO_2_. Renal fibroblasts were pre-stimulated with TMAO for 2 h prior to TNF-α stimulation during the combination treatments. After stimulation, total collagen production was evaluated using Sirius red staining (Thermo fisher Scientific). The renal fibroblasts were incubated with 1 mg/ml Sirius red (diluted in picric acid) for 30 min at room temperature. The fibroblasts were then washed with PBS and destained with NaOH 0.1 M on a shaker at 700 rpm for 15 min. The destaining solutions were transferred to a new 96-well plate and evaluated at 540 nm using the Cytation 3 plate reader.

### Targeted protein analysis

The renal fibroblasts were stimulated with TMAO (300 µM), TNF-α (1, 10 or 50 ng/ml) or the combination of both for 24 h and incubated at 37ºC with 5% CO_2_. Renal fibroblasts were pre-stimulated with TMAO for 2 h prior to TNF-α stimulation during the combination treatments. The renal fibroblast supernatants were analysed using the proximity extension assay (PEA) technology. In short, a set of antibodies labelled with oligonucleotides are allowed to target proteins. When these antibodies come close to each other, they create a PCR target sequence, which is detected and quantified using standard real-time PCR. The Olink inflammation panel (Olink Bioscience AB, Uppsala, Sweden) utilizes PEA technology, allowing the examination of 92 inflammation-related proteins. The recorded protein values are presented as linearized normalized protein expression levels (NPX). Proteins with signals lower than the limit of detection (LOD) were excluded from further analysis.

### Data analysis

All data are expressed as mean ± SEM. The differences between the groups were analyzed by one-way ANOVA followed by Bonferroni multiple testing correction. Differences were considered statistically significant at *p* < 0.05.

## Results

### TMAO and TNF-α synergistically increase fibronectin release from renal fibroblasts

**Figure 1:**
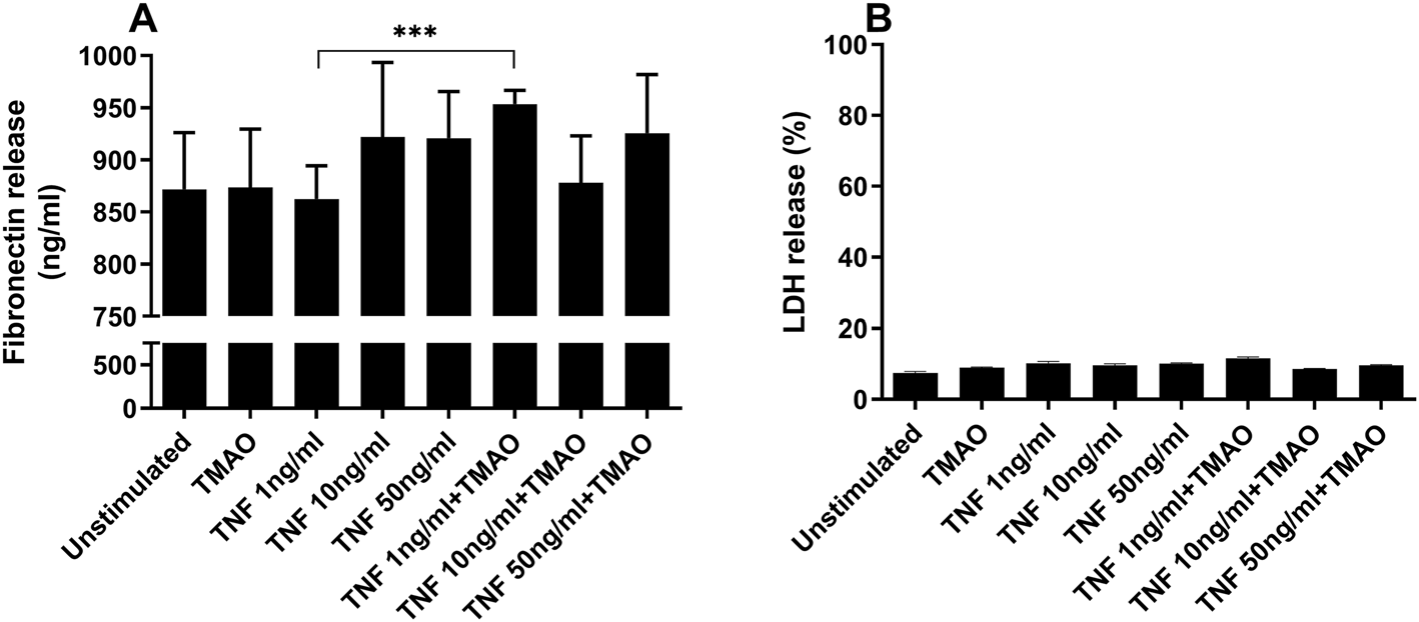
TNF-α and TMAO have a synergistic effect on fibronectin release. Renal fibroblasts were stimulated with TMAO 300 μM and TNF-α (1, 10, 50 ng/ml ) alone or in combination for 24 h (**A**) or 48 h (**B**) and fibronectin release (**A**) and cell viability was evaluated (**B**). LDH release is presented as % of total LDH. Data are presented as mean ± SEM (*n* = 4 independent experiments). Asterisks denote statistical significance compared to unstimulated cells (*p < 0.05, ***p < 0.001).

We started by investigating whether the fibrotic effect of TNF-α on renal fibroblasts can be enhanced by TMAO. In accordance with our previous findings^22^, we found no significant difference in fibronectin release from renal fibroblasts stimulated with TMAO 300 μM compared to unstimulated cells after 24 h (Fig. 1A). Similarly, TNF-α stimulation caused no change in fibronectin release compared to unstimulated cells (Fig. 1A). Interestingly, the combination treatment of TNF-α 1 ng/ml and TMAO synergistically increased fibronectin release in comparison to TNF-α 1 ng/ml alone (Fig. 1A). Renal fibroblasts exhibited no decreased cell viability (LDH release) after 48 h treatment with TMAO or TNF-α alone or in combination (Fig. 1B). Our results suggest that the combined exposure of TMAO and TNF-α 1 ng/ml can increase fibronectin release from renal fibroblasts, independent of cell death.

### TMAO and TNF-α promote renal fibroblast proliferation via Akt/mTOR and ERK pathways

**Figure 2:**
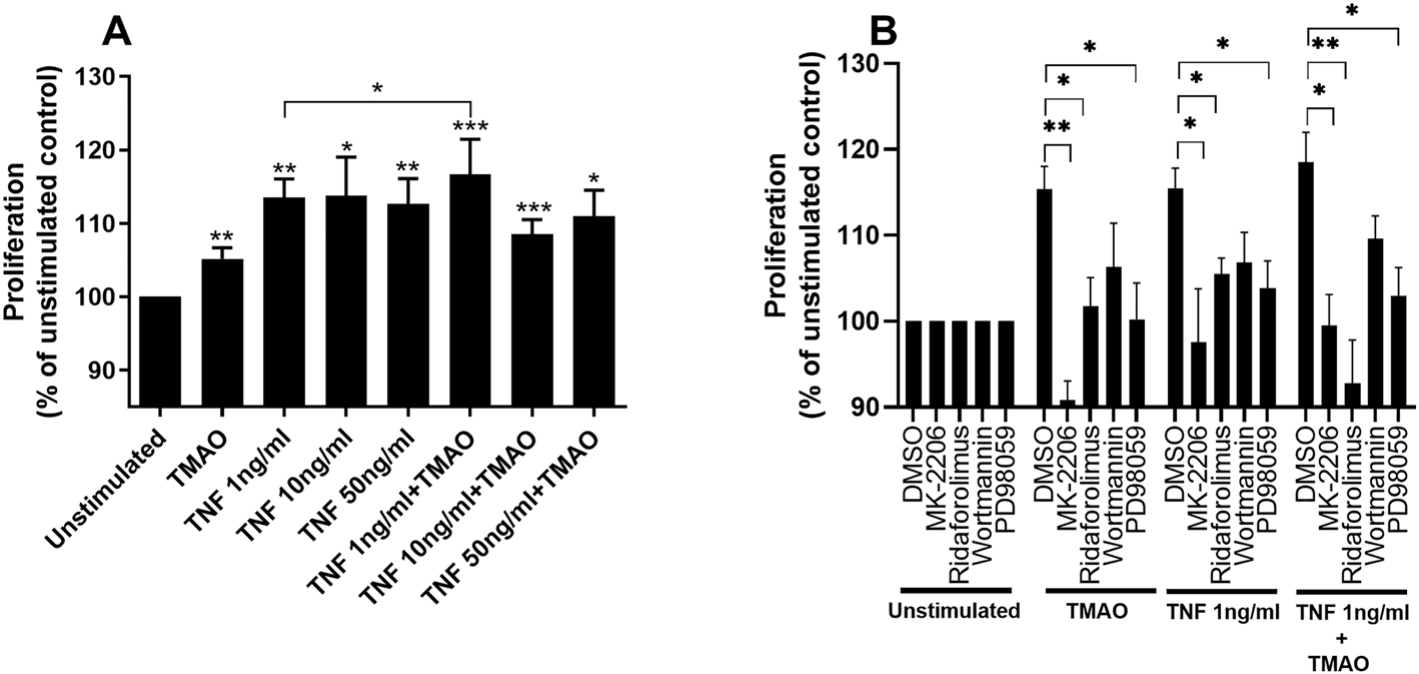
TNF-α and TMAO increase renal fibroblast proliferation. Renal fibroblasts were stimulated with TMAO 300 μM and TNF-α (1, 10, 50 ng/ml) alone or in combination for 48 h and proliferation was evaluated (**A**). Renal fibroblasts were also pre-incubated with DMSO (vehicle), Akt inhibitor MK-2206 (1 µM), mTOR inhibitor ridaforolimus (1 µM), PI3K inhibitor wortmannin (1 µM) or ERK inhibitor PD98059 (1uM) for 1 h prior to stimulation for 48 h (**B**) followed by proliferation evaluation. Proliferation is presented as % of unstimulated control. Data are presented as mean ± SEM (*n* = 8–10 independent experiments). Asterisks denote statistical significance compared to unstimulated cells (*p < 0.05, **p < 0.01, ***p < 0.001).

Next, we wanted to elucidate whether the combination of TMAO and TNF-α could increase renal fibroblast proliferation. We found that TMAO 300 μM and TNF-α 1 ng/ml alone or in combination, significantly increased renal fibroblast proliferation compared to unstimulated cells after 48 h (Fig. 2A). Notably, the combination of TNF-α 1 ng/ml and TMAO caused a significant increased proliferation compared to TNF-α 1 ng/ml alone (Fig. 2A). However, this increase was not synergistic. Next, we investigated which signaling pathways mediate the proliferative effect of TMAO and TNF-α on renal fibroblasts. We found that inhibition of Akt (MK-2206), mTOR (Ridaforolimus), ERK (PD98059), but not PI3K (Wortmannin), resulted in reduced cell proliferation after stimulation with TMAO and TNF-α alone or in combination compared to DMSO treated cells after 48 h (Fig. 2B). Taken together, our results show that TMAO and TNF-α mediate their proliferative effect on renal fibroblast using the Akt/mTOR and ERK signaling pathways.

### TMAO and TNF-α synergistically increases total collagen production via Akt/mTOR and ERK pathways

**Figure 3:**
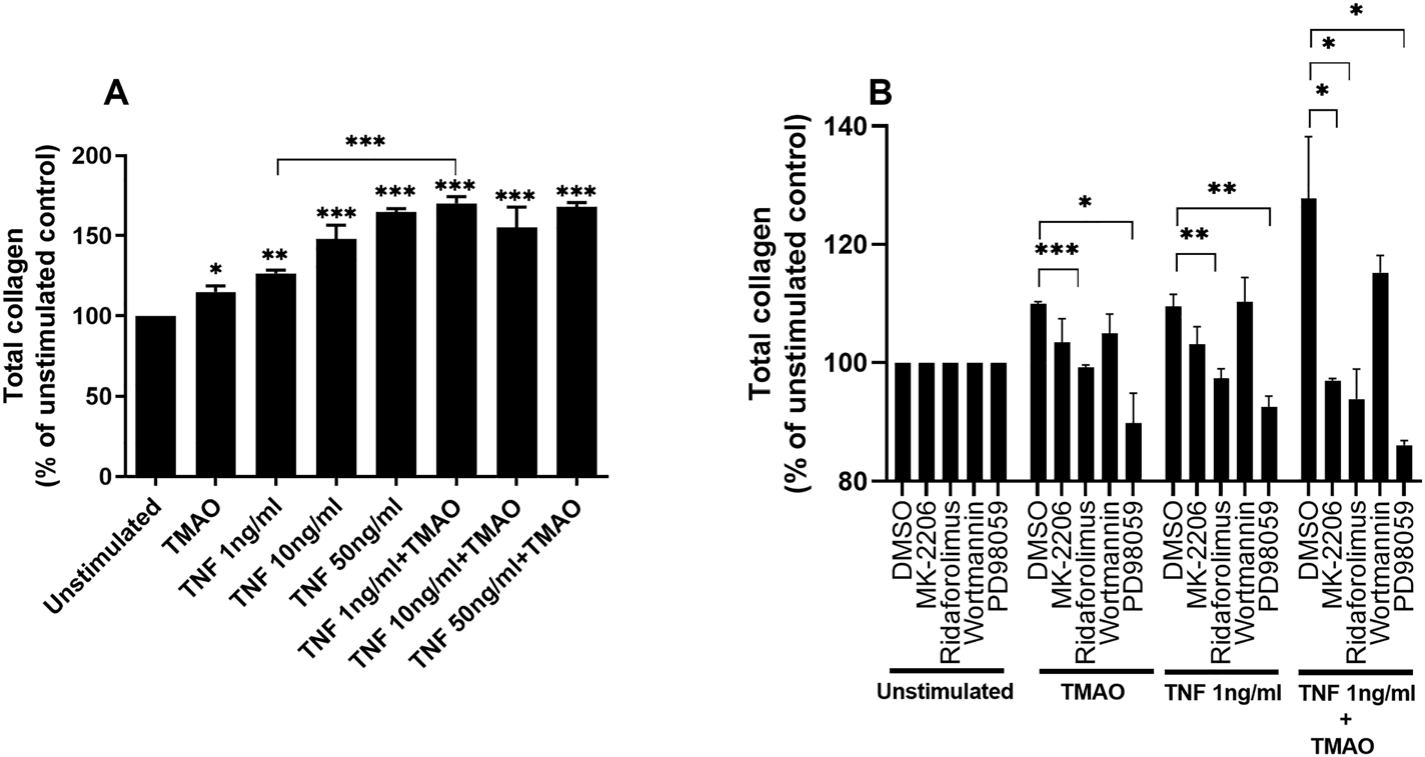
TNF-α and TMAO have a synergistic effect on total collagen production. Renal fibroblasts were stimulated with TMAO 300 μM and TNF-α (1, 10, 50 ng/ml) alone or in combination for 96 h and total collagen production was evaluated (**A**). Renal fibroblasts were also pre-incubated with DMSO (vehicle), Akt inhibitor MK-2206 (1 µM), mTOR inhibitor ridaforolimus (1 µM), PI3K inhibitor wortmannin (1 µM) or ERK inhibitor PD98059 (1uM) for 1 h prior to stimulation for 48 h (**B**) followed by total collagen evaluation. Total collagen is presented as % of unstimulated control. Data are presented as mean ± SEM (*n* = 4 independent experiments). Asterisks denote statistical significance compared to unstimulated cells (*p < 0.05, **p < 0.01, ***p < 0.001).

We continued to investigate whether TMAO could enhance TNF-α mediated total collagen production from renal fibroblasts. We found that TMAO 300 μM and TNF-α alone or in combination increased total collagen production compared to unstimulated cells (Fig. 3A). We also found that the combination treatments of TNF-α 1 ng/ml and TMAO significantly induced a synergistic increase in total collagen production compared to TNF-α 1 ng/ml alone (Fig. 3A). Moreover, we found that inhibition of Akt (MK-2206), mTOR (Ridaforolimus) and ERK (PD98059), but not PI3K (Wortmannin), resulted in reduced total collagen production after stimulation with TMAO and TNF-α 1 ng/ml compared to DMSO treated cells after 96 h (Fig. 3B). Our findings suggest that the combination of TNF-α 1 ng/ml and TMAO synergistically increases total collagen production via Akt/mTOR and ERK pathways.

### TNF-α and TMAO synergistically increase the release of cytokines from renal fibroblasts

**Figure 4:**
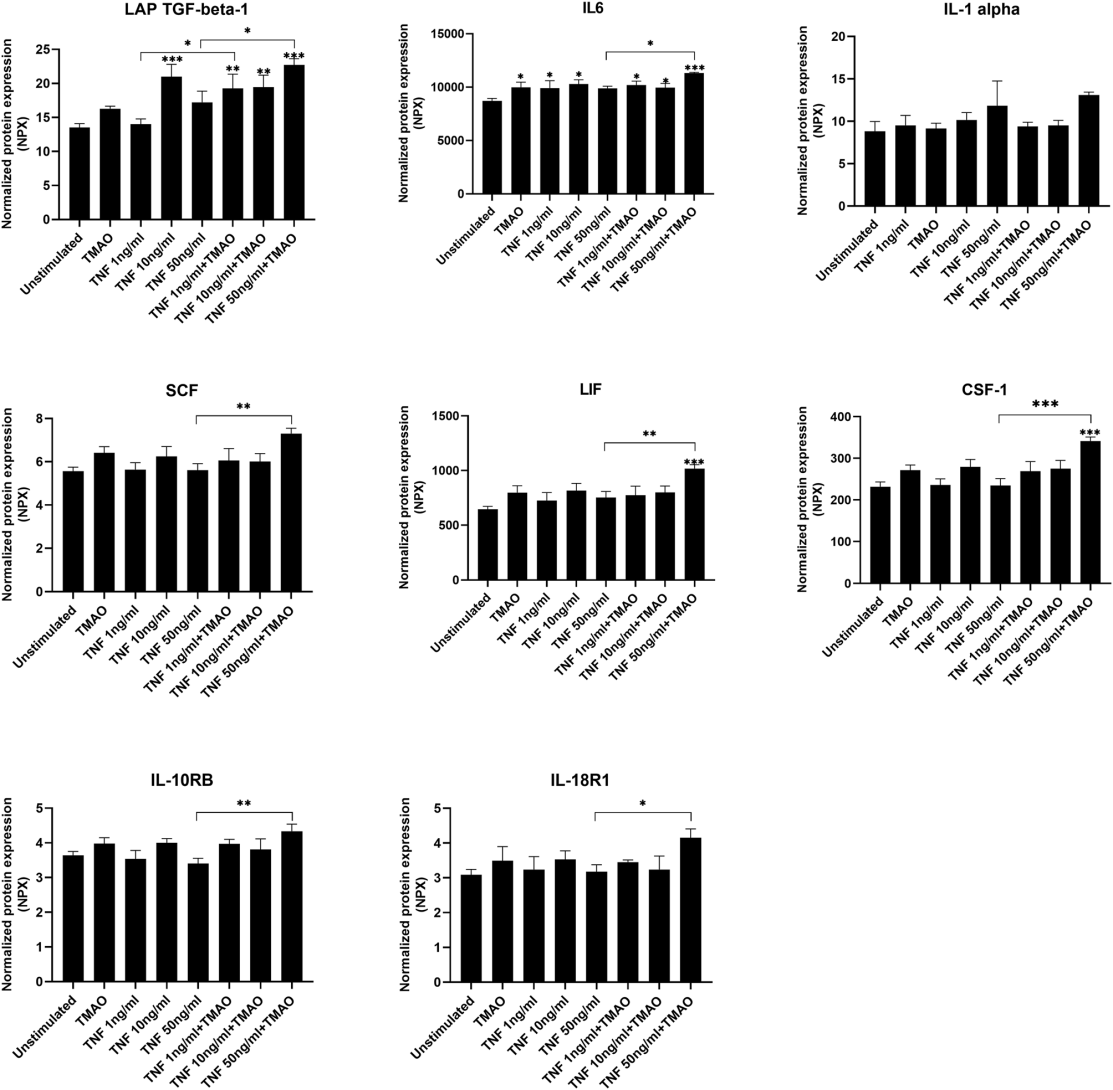
Secretion of cytokines from renal fibroblasts. Renal fibroblasts were stimulated with TMAO 300 μM and TNF-α (1, 10, 50 ng/ml) alone or in combination for 24 h followed by Olink targeted protein analysis. The data are presented as linearized NPX values. Data are presented as mean ± SEM (*n* = 4 independent experiments). Asterisks denote statistical significance compared to unstimulated cells (*p < 0.05, **p < 0.01, ***p < 0.001).

Next, we continued to evaluate whether TMAO could enhance TNF-α mediated cytokine /cytokine receptor release from renal fibroblasts using a targeted protein analysis. We found that TMAO 300 μM and TNF-α alone increased the release of IL-6 compared to unstimulated cells after 24 h (Fig. 4). TNF-α was also found to increase LAP TGF-β-1 release compared to unstimulated cells (Fig. 4). We found that the combination of TNF-α 50 ng/ml and TMAO significantly enhanced the release of LAP TGF-β-1, IL-6, SCF, LIF, CSF-1, IL-10RB and IL-18R1 compared to TNF-α 50 ng/ml alone (Fig. 4). LAP TGF-β-1 release was also significantly increased after TNF-α 1 ng/ml and TMAO stimulation compared to TNF-α 1 ng/ml alone (Fig. 4). Taken together, our results suggest that the combination of TNF-α and TMAO can enhance the release of cytokines from renal fibroblasts.

### TMAO and TNF-α synergistically increase the secretion of chemokines

**Figure 5:**
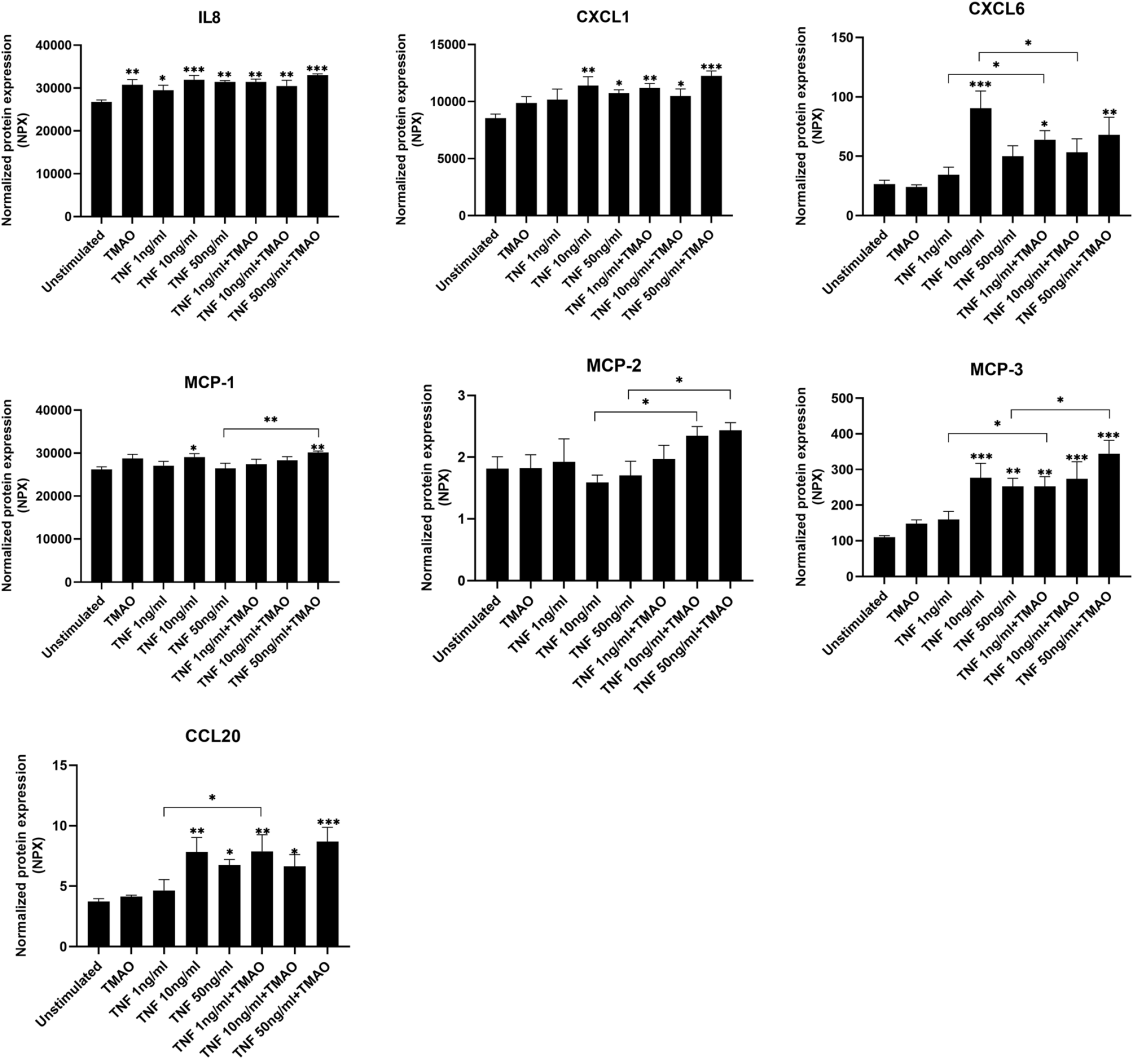
Chemokine release from renal fibroblasts. Renal fibroblasts were stimulated with TMAO 300 μM and TNF-α (1, 10, 50 ng/ml) alone or in combination for 24 h followed by Olink targeted protein analysis. The data are presented as linearized NPX values. Data are presented as mean ± SEM (*n* = 4 independent experiments). Asterisks denote statistical significance compared to unstimulated cells (*p < 0.05, **p < 0.01, ***p < 0.001).

In the next step we also wanted to evaluate whether TMAO could enhance TNF-α mediated chemokine release from renal fibroblasts. We found that TMAO 300 μM alone increased the release of IL-8 compared to unstimulated cells after 24 h (Fig. 5). TNF-α was found to increase IL-8, CXCL-1, CXCL-6, MCP-1, MCP-3 and CCL20 release compared to unstimulated cells (Fig. 5). In addition, we found that the combination of TNF-α 50 ng/ml and TMAO significantly enhanced the release of MCP-1, MCP-3 and MCP-2 compared to TNF-α 50 ng/ml alone (Fig. 5). Moreover, the combination of TNF-α 10 ng/ml and TMAO, significantly increased the secretion of MCP-2 compared to TNF-α 10 ng/ml alone. The combination of TNF-α 1 ng/ml and TMAO also increased the release of CXCL-6, MCP-3 and CCL20 compared to TNF-α 1 ng/ml alone (Fig. 5). Hence, our results suggest that the combination of TNF-α and TMAO can enhance the release of several chemokines from renal fibroblasts.

### TMAO enhances TNF-α mediated release of inflammatory-and growth mediators

**Figure 6:**
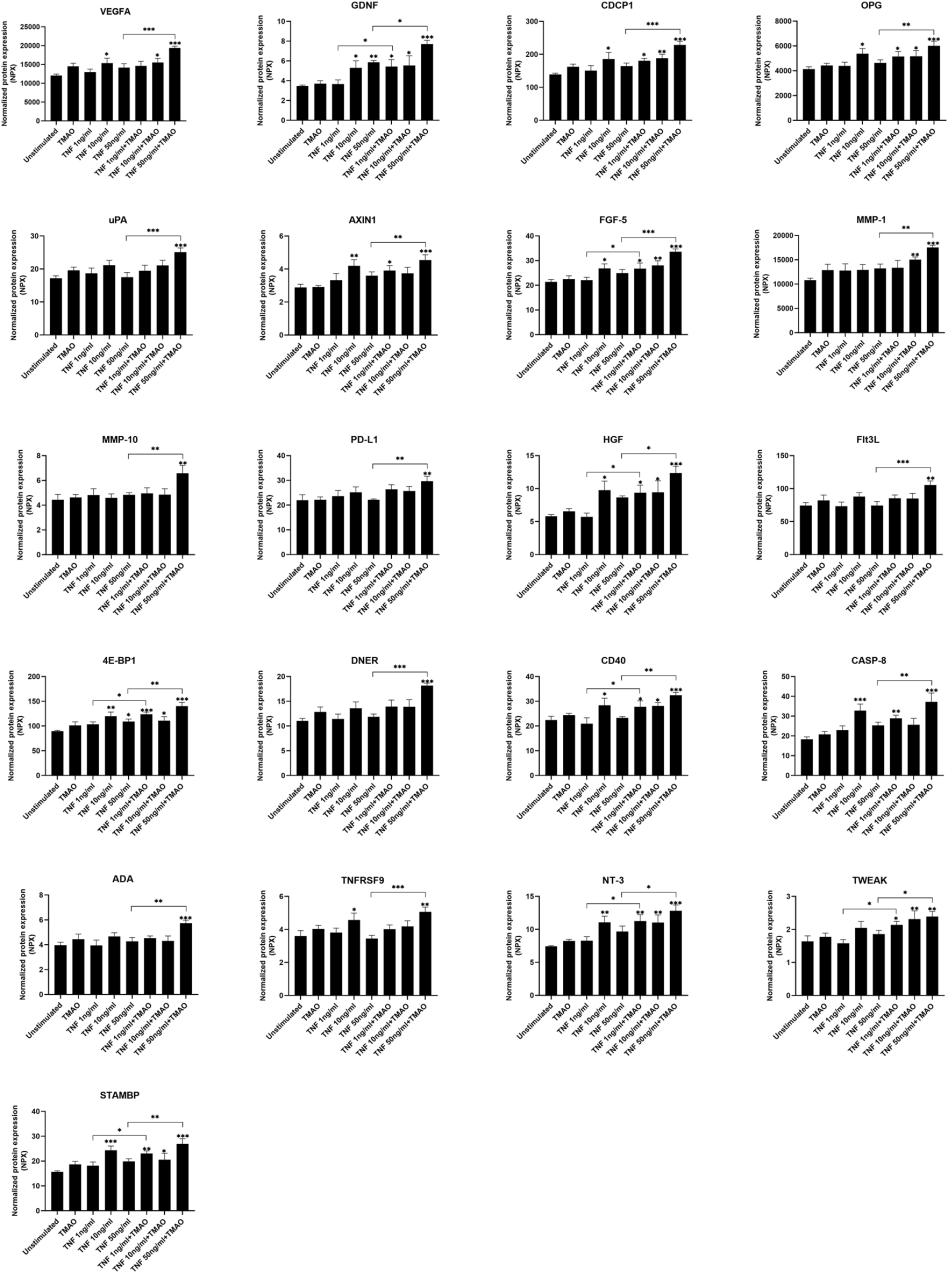
Secretion of inflammatory-and growth mediators from renal fibroblasts. Renal fibroblasts were stimulated with TMAO 300 μM and TNF-α (1, 10, 50 ng/ml) alone or in combination for 24 h followed by Olink targeted protein analysis. The data are presented as linearized NPX values. Data are presented as mean ± SEM (*n* = 4 independent experiments). Asterisks denote statistical significance compared to unstimulated cells (*p < 0.05, **p < 0.01, ***p < 0.001).

Next, we continued to investigate whether TMAO could enhance TNF-α mediated release of additional inflammatory-and growth mediators from renal fibroblasts. We found that TNF-α alone, but not TMAO, significantly increased the release of VEGFA, GDNF, CDCP1, OPG, AXIN1, FGF-5, HGF, 4E-BP1, CD40, CASP-8, TNFRSF9, NT-3, STAMBP compared to unstimulated cells (Fig. 6). We also found that the release of VEGFA, GDNF, CDCP1, OPG, uPA, AXIN1, FGF-5, MMP-1, MMP-10, PD-L1, HGF, Flt3L, 4E-BP1, DNER, CD40, CASP-8, ADA, TNFRSF9, NT-3, TWEAK and STAMBP were enhanced by the combination of TNF-α 50 ng/ml and TMAO compared to TNF-α 50 ng/ml alone (Fig. 6). Furthermore, the release of GDNF, FGF-5, HGF, 4E-BP1, CD40, NT-3, TWEAK and STAMBP were enhanced by the combination of TNF-α 1 ng/ml and TMAO compared to TNF-α 1 ng/ml alone (Fig. 6). Taken together, our results suggest that TNF-α and TMAO enhances the release of several inflammatory-and growth mediators from renal fibroblasts.

## Discussion

Several studies indicate that TMAO exacerbates tubulointerstitial fibrosis and renal inflammation in CKD^6,21–23^. Furthermore, there is substantial evidence demonstrating that TNF-α plays a significant role in contributing to renal fibrosis^18,19^. However, the additive or synergistic contribution of TMAO and TNF-α on renal inflammation and fibrosis is not well understood. Our aim was therefore to investigate if TMAO can enhance the inflammatory and fibrotic effects of TNF-α on renal fibroblast and to elucidate the molecular pathways involved.

We started by evaluating the effect of TMAO and TNF-α on fibronectin release from renal fibroblasts. We found that neither TMAO nor TNF-α increased fibronectin release from renal fibroblasts. This is in accordance with our previous findings^22^. However, we found that the combination of TMAO and TNF-α can increase fibronectin release compared to TNF-α alone. The observed fibronectin release was independent of fibroblast cell death. We have previously shown that TMAO is unable to induce increased fibronectin release from renal fibroblast up to 96 h posttreatment^22^. TMAO and TNF-α, independent of concentration, did not mediate increased fibroblast cell death. Fibronectin, a high molecular weight glycoprotein with adhesive properties, holds a pivotal function in both wound-healing processes and the formation of extracellular matrix^27^. Under pathophysiological conditions, fibronectin expression levels are dramatically increased in the renal tubulointerstitium which contributes to renal fibrosis. Taken together, our findings indicate that the combined exposure of TMAO and TNF-α can increase fibronectin release from renal fibroblasts.

We next evaluated the effect of TMAO and TNF-α on renal fibroblast proliferation and collagen production. We found that both TNF-α and TMAO induced increased fibroblast proliferation and that the combination of TNF-α 1 ng/ml and TMAO induced increased cell proliferation compared to TNF-α alone. However, this increase was more additive than synergistic. Furthermore, the overall difference between the treatment groups was very small, which strengthens the notion of an additive effect. We also found that TMAO and TNF-α alone or in combination increased total collagen production. The combination treatments of TNF-α 1 ng/ml and TMAO synergistically increased total collagen production compared to TNF-α alone. Next, we evaluated which signaling pathways TMAO and TNF-α activate to induce proliferation and collagen production. This was done to evaluate the mechanism behind the observed synergistic effects of TMAO and TNF-α. Our findings showed that both TMAO and TNF-α mediate their proliferative and collagen inducing effects on renal fibroblast via Akt, mTOR and ERK, but not PI3K. We have previously shown that TMAO induced increased renal fibroblast proliferation and collagen production via Akt and mTOR, but not via PI3K^22^. We have also shown that TMAO can reduce megalin expression in proximal tubular cells via PI3K and ERK^28^. In addition, TMAO has been linked to promote vascular inflammation via ERK activation^29^. TNF-α is also known to activate Akt, mTOR, and ERK in different cells^20,30,31^. Hence, the synergistic effects of TMAO and TNF-α could be explained by their ability to activate the same pathways. Taken together, our findings indicate that Akt, mTOR and ERK, but not PI3K, mediates the effect of TMAO and TNF-α on fibroblast proliferation and collagen production.

We continued to investigate whether TMAO could enhance TNF-α mediated release of inflammatory mediators from renal fibroblasts using Olink multiplex assay including 92 proteins. We found that TMAO could enhance TNF-α mediated release of a variety of cytokines/cytokine receptors LAP TGFβ-1, IL-6 SCF, LIF, CSF-1, IL-10RB and IL-18R1 from renal fibroblasts compared TNF-α alone. Each of these cytokines have been shown to play a role in the pathophysiology of kidney disease^32–38^. TGF-β1 is known to promote myofibroblast activation and induce the expression of fibronectin and collagen^32^. IL-6 has also been found to induce tubular atrophy, increase collagen production and accelerate tubulointerstitial fibrosis^33^. Interestingly, TMAO was also able to significantly induce IL-6 release alone from renal fibroblast, strengthening the link to renal fibrosis. Moreover, TMAO was also found to enhance TNF-α mediated secretion of several chemokines CXCL-6, MCP-1, MCP-2, MCP-3 and CCL20 compared to TNF-α alone. All these chemokines participate in the pathogenesis of kidney disease according to previous publications^39–43^. Inhibition of MCP-1 in renal disease has been shown to lead to patient improvements^40^. CCL20 has also been found to be increased in kidney disease. CCL20 promotes T-cell recruitment, renal tissue injury and reduced renal function^43^. We also found, using the Olink proteomic platform, that TNF-α and TMAO enhanced the secretion of additional inflammatory-and growth mediators associated with kidney disease; VEGFA, GDNF, CDCP1, OPG, uPA, AXIN1, MMP-1, MMP-10, PD-L1, HGF, Flt3L, 4E-BP1, CD40, CASP-8, ADA, TNFRSF9, TWEAK^44–61^. Increased systemic levels of TWEAK has been shown to trigger kidney injury, inflammation, and renal fibrosis. The Ras/ERK/ NFκB pathways play a crucial role in TWEAK-induced fibroblast proliferation and inflammation^61^. Circulating levels of sCD40 and sCD40L have also been shown to be associated with renal injury^56^. Taken together, our results suggest that the TMAO can enhance TNF-α mediated kidney inflammation by inducing the release of several cytokines, chemokines, inflammatory-and growth mediators from renal fibroblasts.

Our study is the first aiming to elucidate the combined effect of TMAO and TNF-α on renal fibroblasts through assessing aspects of renal fibrosis and inflammation. The current existing research has focused mainly on how TMAO or TNF-α alone affects the progress of kidney disease. However, the co-existence of TMAO and TNF-α (and/or other pro-inflammatory factors) in the renal interstitium is closer to the pathophysiologic background of kidney disease.

In conclusion, our findings showed that TMAO enhances TNF-α mediated renal fibroblast proliferation and collagen production via Akt/mTOR/ERK signaling pathway. We also observed that the combination of TMAO and TNF-α increased the release of several inflammatory mediators associated with kidney disease. To the best of our knowledge, this is the first study elucidating the synergistic effects of TMAO and TNF-α on renal inflammation and fibrosis. Our results can promote further research evaluating the combined effect of TMAO and inflammatory mediators on the development of kidney disease.

## Data Availability

All data generated or analysed during this study are included in this published article.

## References

[1] Velasquez MT, Ramezani A, Manal A, Raj DS (2016). Trimethylamine N-Oxide: The good, the bad and the unknown. Toxins Basel.

[2] Zixin Y (2022). TMAO as a potential biomarker and therapeutic target for chronic kidney disease: A review. Front. Pharmacol..

[3] Miao L, Du J, Chen Z, Shi D, Qu H (2021). Effects of microbiota-driven therapy on circulating trimethylamine-N-oxide metabolism: A systematic review and meta-analysis. Front Cardiovasc. Med..

[4] Ma J, Pazos IM, Gai F (2014). Microscopic insights into the protein-stabilizing effect of trimethylamine N-oxide (TMAO). Proc. Natl. Acad. Sci. USA.

[5] Ufnal M, Zadlo A, Ostaszewski R (2015). TMAO: A small molecule of great expectations. Nutrition.

[6] Tang WH (2015). Gut microbiota-dependent trimethylamine N-oxide (TMAO) pathway contributes to both development of renal insufficiency and mortality risk in chronic kidney disease. Circ. Res..

[7] Mueller DM (2015). Plasma levels of trimethylamine-N-oxide are confounded by impaired kidney function and poor metabolic control. Atherosclerosis.

[8] Pelletier CC (2019). Elevation of trimethylamine-N-oxide in chronic kidney disease: Contribution of decreased glomerular filtration rate. Toxins Basel.

[9] Lau WL, Savoj J, Nakata MB, Vaziri ND (2018). Altered microbiome in chronic kidney disease: Systemic effects of gut-derived uremic toxins. Clin. Sci. Lond..

[10] Gruppen EG (2017). TMAO is associated with mortality: Impact of modestly impaired renal function. Sci. Rep..

[11] Kim RB (2016). Advanced chronic kidney disease populations have elevated trimethylamine N-oxide levels associated with increased cardiovascular events. Kidney Int..

[12] Tomlinson JAP, Wheeler DC (2017). The role of trimethylamine N-oxide as a mediator of cardiovascular complications in chronic kidney disease. Kidney Int..

[13] Rodríguez-Iturbe B, García García G (2010). The role of tubulointerstitial inflammation in the progression of chronic renal failure. Nephron. Clin. Pract..

[14] Nast CC (2017). The renal tubulointerstitium. Adv Chronic Kidney Dis.

[15] Liu Y (2011). Cellular and molecular mechanisms of renal fibrosis. Nat. Rev. Nephrol..

[16] Sato Y, Yanagita M (2017). Resident fibroblasts in the kidney: A major driver of fibrosis and inflammation. Inflamm. Regen..

[17] Hodgkins KS, Schnaper HW (2012). Tubulointerstitial injury and the progression of chronic kidney disease. Pediatr. Nephrol..

[18] Taguchi S (2021). Effects of tumor necrosis factor-α inhibition on kidney fibrosis and inflammation in a mouse model of aristolochic acid nephropathy. Sci. Rep..

[19] Therrien FJ, Agharazii M, Lebel M, Larivière R (2012). Neutralization of tumor necrosis factor-alpha reduces renal fibrosis and hypertension in rats with renal failure. Am. J. Nephrol..

[20] Lousa I, Reis F, Santos-Silva A, Belo L (2022). The signaling pathway of TNF receptors: Linking animal models of renal disease to human CKD. Int. J. Mol. Sci..

[21] Zhang W (2021). Inhibition of microbiota-dependent TMAO production attenuates chronic kidney disease in mice. Sci. Rep..

[22] Kapetanaki S, Kumawat AK, Persson K, Demirel I (2021). The fibrotic effects of TMAO on human renal fibroblasts is mediated by NLRP3, caspase-1 and the PERK/Akt/mTOR pathway. Int. J. Mol. Sci..

[23] Fang Q (2021). Trimethylamine N-oxide exacerbates renal inflammation and fibrosis in rats with diabetic kidney disease. Front Physiol..

[24] Constantino-Jonapa LA (2023). Contribution of trimethylamine N-oxide (TMAO) to chronic inflammatory and degenerative diseases. Biomedicines.

[25] Liu Y, Dai M (2020). Trimethylamine N-oxide generated by the gut microbiota is associated with vascular inflammation: New insights into atherosclerosis. Mediat. Inflamm..

[26] Muller GA, Frank J, Rodemann HP, Engler-Blum G (1995). Human renal fibroblast cell lines (tFKIF and tNKF) are new tools to investigate pathophysiologic mechanisms of renal interstitial fibrosis. Exp. Nephrol..

[27] Bülow RD, Boor P (2019). Extracellular matrix in kidney fibrosis: More than just a scaffold. J. Histochem. Cytochem..

[28] Kapetanaki S, Kumawat AK, Persson K, Demirel I (2022). TMAO suppresses megalin expression and albumin uptake in human proximal tubular cells via PI3K and ERK signaling. Int. J. Mol. Sci..

[29] Seldin MM (2016). Trimethylamine N-Oxide promotes vascular inflammation through signaling of mitogen-activated protein kinase and nuclear factor-κB. J. Am. Heart Assoc..

[30] Gao ZG, Zuberi A, Quon MJ, Dong ZG, Ye JP (2003). Aspirin inhibits serine phosphorylation of insulin receptor substrate 1 in tumor necrosis factor-treated cells through targeting multiple serine kinases. J. Biol. Chem..

[31] Xu C (2022). TNFα and IFNγ rapidly activate PI3K-AKT signaling to drive glycolysis that confers mesenchymal stem cells enhanced anti-inflammatory property. Stem Cell Res. Ther..

[32] Sureshbabu A, Muhsin SA, Choi ME (2016). TGF-β signaling in the kidney: Profibrotic and protective effects. Am. J. Physiol. Renal. Physiol..

[33] Su H, Lei CT, Zhang C (2017). Interleukin-6 signaling pathway and its role in kidney disease: An update. Front. Immunol..

[34] El-Koraie AF, Baddour NM, Adam AG, El Kashef EH, El Nahas AM (2001). Role of stem cell factor and mast cells in the progression of chronic glomerulonephritides. Kidney Int..

[35] Xu S (2022). Leukemia inhibitory factor is a therapeutic target for renal interstitial fibrosis. EBioMedicine.

[36] Jang MH (2006). Distinct in vivo roles of colony-stimulating factor-1 isoforms in renal inflammation. J. Immunol..

[37] Wei W, Zhao Y, Zhang Y, Jin H, Shou S (2022). The role of IL-10 in kidney disease. Int. Immunopharmacol..

[38] Thomas JM (2021). IL-18 (Interleukin-18) produced by renal tubular epithelial cells promotes renal inflammation and injury during deoxycorticosterone/salt-induced hypertension in mice. Hypertension.

[39] Sun MY (2019). CXCL6 Promotes renal interstitial fibrosis in diabetic nephropathy by activating JAK/STAT3 signaling pathway. Front Pharmacol..

[40] Haller H, Bertram A, Nadrowitz F, Menne J (2016). Monocyte chemoattractant protein-1 and the kidney. Curr. Opin. Nephrol. Hypertens..

[41] Gonzalez J (2013). Dual effect of chemokine CCL7/MCP-3 in the development of renal tubulointerstitial fibrosis. Biochem. Biophys. Res. Commun..

[42] Lee J (2022). Chemokine (C-C Motif) ligand 8 and tubulo-interstitial injury in chronic kidney disease. Cells.

[43] Lebherz-Eichinger D (2014). Increased chemokine excretion in patients suffering from chronic kidney disease. Transl. Res..

[44] Doi K, Noiri E, Fujita T (2010). Role of vascular endothelial growth factor in kidney disease. Curr. Vasc. Pharmacol..

[45] Michos O (2010). Kidney development in the absence of Gdnf and Spry1 requires Fgf10. PLoS Genet.

[46] Kajiwara K (2021). CDCP1 promotes compensatory renal growth by integrating Src and Met signaling. Life Sci. Alliance..

[47] Kamińska J (2021). Circulating osteoprotegerin in chronic kidney disease and all-cause mortality. Int. J. Gen. Med..

[48] Zhang G, Eddy AA (2008). Urokinase and its receptors in chronic kidney disease. Front. Biosci..

[49] Malik SA, Modarage K, Goggolidou P (2020). The role of wnt signalling in chronic kidney disease (CKD). Genes Basel.

[50] Zakiyanov O, Kalousová M, Zima T, Tesař V (2019). Matrix metalloproteinases in renal diseases: A critical appraisal. Kidney Blood Press Res..

[51] Sun X, Liu Y (2022). Matrix metalloproteinase-10 in kidney injury repair and disease. Int. J. Mol. Sci..

[52] Curran CS, Kopp JB (2021). PD-1 immunobiology in glomerulonephritis and renal cell carcinoma. BMC Nephrol..

[53] Liu Y (2004). Hepatocyte growth factor in kidney fibrosis: Therapeutic potential and mechanisms of action. Am. J. Physiol. Renal. Physiol..

[54] Evers BD (2016). CD103+ Kidney dendritic cells protect against crescentic GN by maintaining IL-10-producing regulatory T cells. J. Am. Soc. Nephrol..

[55] Holditch SJ (2019). The consequences of increased 4E-BP1 in polycystic kidney disease. Hum Mol Genet.

[56] Zhang S, Breidenbach JD, Russell BH, George J, Haller ST (2020). CD40/CD40L Signaling as a promising therapeutic target for the treatment of renal disease. J. Clin. Med..

[57] Kaushal GP, Basnakian AG, Shah SV (2004). Apoptotic pathways in ischemic acute renal failure. Kidney Int..

[58] Lu CF, Liu WS, Ge XQ, Xu F (2021). Serum adenosine deaminase levels are associated with diabetic kidney disease in type 2 diabetic patients. Endocr. Connect..

[59] Nano J (2022). Novel biomarkers of inflammation, kidney function and chronic kidney disease in the general population. Nephrol. Dial. Transplant..

[60] Sanz AB (2014). TWEAK and the progression of renal disease: Clinical translation. Nephrol. Dial Trans..

[61] Ucero AC (1832). TNF-related weak inducer of apoptosis (TWEAK) promotes kidney fibrosis and Ras-dependent proliferation of cultured renal fibroblast. Bba-Mol. Basis Dis..

